# Synthesis and Biological Evaluation of 2-Aminobenzamide Derivatives as Antimicrobial Agents: Opening/Closing Pharmacophore Site

**DOI:** 10.3390/ijms15035115

**Published:** 2014-03-21

**Authors:** Yahia N. Mabkhot, Abdullah M. Al-Majid, Assem Barakat, Salim S. Al-Showiman, Munirah S. Al-Har, Smaail Radi, Muhammad Moazzam Naseer, Taibi B. Hadda

**Affiliations:** 1 Department of Chemistry, College of Science, King Saud University, P. O. Box 2455, Riyadh 11451, Saudi Arabia; E-Mails: amajid@ksu.edu.sa (A.M.A.-M.); ambarakat@ksu.edu.sa (A.B.); showiman@ksu.edu.sa (S.S.A.-S.); 2 Department of Chemistry, Faculty of Science, Alexandria University, P. O. Box 426, Ibrahimia 21321, Alexandria, Egypt; 3 Department of Chemistry, College of Sciences, Hail University, P. O. Box 2440, Hail 81451, Saudi Arabia; E-Mail: mun_eera2010@hotmail.com; 4 LCAE, Faculté des Sciences, Université Mohammed Premier, Oujda BP 717 60000, Morocco; E-Mail: radi_smaail@yahoo.fr; 5 Department of Chemistry, Quaid-i-Azam University, Islamabad 45320, Pakistan; E-Mail: muhammadmoazzamnaseer@gmail.com; 6 Laboratoire Chimie Matériaux, Faculté Sciences, Université Mohammed Premier, Oujda BP 717 60000, Morocco; E-Mail: taibi.ben.hadda@gmail.com

**Keywords:** isatoic anhydride, conventional method, microwave method, 2-aminobenzamide, antimicrobial activity

## Abstract

A series of new 2-aminobenzamide derivatives (**1**–**10**) has been synthesized in good to excellent yields by adopting both conventional and/or a time-efficient microwave assisted methodologies starting from isatoic anhydride (ISA) and characterized on the basis of their physical, spectral and microanalytical data. Selected compounds of this series were then tested against various bacterial (*Bacillus subtilis* (RCMB 000107) and *Staphylococcus aureus* (RCMB 000106). *Pseudomonas aeruginosa* (RCMB 000102) and *Escherichia coli* (RCMB 000103) and fungal strains (*Saccharomyces cerevisiae* (RCMB 006002), *Aspergillus fumigatus* (RCMB 002003) and *Candida albicans* (RCMB 005002) to explore their potential as antimicrobial agents. Compound **5** was found to be the most active compound among those tested, which showed excellent antifungal activity against *Aspergillus fumigatus* (RCMB 002003) more potent than standard Clotrimazole, and moderate to good antibacterial and antifungal activity against most of the other strains of bacteria and fungi. Furthermore, potential pharmacophore sites were identified and their activity was related with the structures in the solution.

## 1. Introduction

Isatoic anhydride (2*H* 3,1–benzoxacin-2,4 (1*H*)-dione, ISA) is a special compound in organic synthesis, first discovered by Friedländer and Wleügel [1] due to its physicochemical properties and special reactivity features which have been very well exploited in the recent past. Its tremendous importance can be exemplified by the presence of a great number of research papers and patents in the chemical literature, especially in the industrial field where it has been applied for manufacturing agrochemicals, desiccants, paints, fragrances and drug products [2,3]. Recently, this compound has been used as an agent for clinical diagnosis in biological research [4] and as starting material for the synthesis of various antimicrobial agents [5]. Most importantly, isatoic anhydrides serves the role of intermediate in the synthesis of a variety of heterocyclic compounds, such as quinazolinones, quinazolones, benzimidazolones, phthalimides, pyrroloquinazolones, quinazolinediones, in addition to its use in the fluorescent labeling of mRNA and tRNA [6–9]. Among the various heterocycles derived from ISA, quinazolones are the most prominent due to their various biological effects including antihypertensive [10], antimicrobial [11–13], antihyperlipidemic [14,15], antioxidant [16], anti-inflammatory [17–19], anticonvulsant [20,21], and anticancer activities [22–25].

**Scheme 1.**
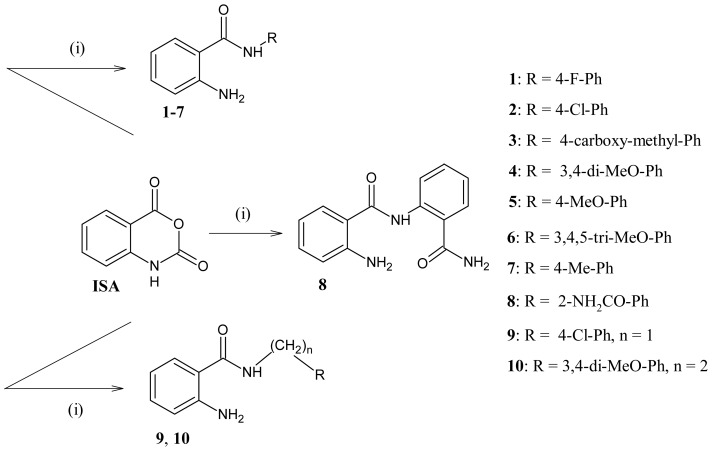
Synthesis of 2-aminobenzamide derivatives (**1**–**10**) from isatoic anhydride (**i**): R-NH_2_ in heating dimethylformamide (DMF).

Owing to the remarkable applications of isatoic anhydride in organic synthesis and medicinal chemistry, we decided to undertake this study. Herein, we report the synthesis of some novel 2-aminobenzamide derivatives by two different methods in good to excellent yields starting from ISA (Scheme 1) and their antimicrobial activity. We found that conventional method (simply heating respective amine with ISA in a suitable solvent) is better than the time-efficient microwave assisted method for the synthesis of such compounds. In antimicrobial screening, compound **5** showed excellent antimicrobial potential, whereas other compounds exhibited low to moderate antimicrobial activity. The factors which affect the activity of these compounds were also identified and reasons for low or moderate activities of certain compounds were explored.

## 2. Results and Discussion

### 2.1. Chemistry

The chemical advantages shown by ISA as precursor for a variety of medicinally important derivatives lie mainly in its capacity to react through two fundamental sites: (a) C8 position of the aromatic ring through electrophilic aromatic substitution or C10 if C8 is occupied by another atom or functional group; and (b) the more electrophilic carbonyl group though nucleophilic acyl substitution reaction generally by losing a CO_2_ group. In addition, it may also react through N atom due to its electronic properties to give some useful products [26].

In the present work, we utilized two different methods, the classical procedure (isatoic anhydride, 1.equiv. plus amine derivatives 1.equiv. in DMF solvent), and the simple, mild and time efficient solvent-free microwave mediated methodology for the synthesis of 2-aminobenzamide derivatives **1**–**10**. Mechanistically, the products **1**–**10** were formed by initial nucleophilic attack of amine derivatives on electrophilic carbonyl group followed by ring opening and elimination of CO_2_ molecules. All the synthesized compounds were fully characterized on the basis of their physical, spectral (IR, NMR, MS) and microanalytical data.

In this context, the synthesis of 2-aminobenzamide derivatives has been proven either by classical method or simple, mild, time efficient, and environment friendly microwave irradiation procedure.

We tried our best to get all compounds of this new series by using the two methods but the microwave method led us to less important yields than conventional method. This is probably due to thermo-sensibility of compounds.

### 2.2. Biological Activity

Selected compounds **1**–**3**, **5** and **7** were screened for their antimicrobial activities. Preliminary screening [27] of the synthesized compounds was performed against three Gram-positive bacteria (*Bacillus subtilis* (RCMB 000107) and *Staphylococcus aureus* (RCMB 000106), two Gram-negative bacteria (*Pseudomonas aeruginosa* (RCMB 000102) and *Escherichia coli* (RCMB 000103), and three fungi (*Saccharomyces cerevisiae* (RCMB 006002), *Aspergillus fumigatus* (RCMB 002003) and *Candida albicans* (RCMB 005002), using the bioassay technique of antibiotics specified in US pharmacopeia at (25 μg/mL). Most of the tested compounds showed moderate to good activity against one or more bacterial and/or fungal strains. However, the compound **5** was found to be the most active as compared to all other tested compounds with excellent antifungal activity (even more than the standard drug Clotrimazole) against *Aspergillus fumigatus* (RCMB 002003) (Table 1). It also showed excellent antifungal activity against *Saccharomyces cerevislae* (RCMB 006002) slightly less than the standard drug and good antibacterial activity against all bacterial strains . The results of the present investigation confirm the tremendous biological potential of compound **5** and suggest that it should be concentrated for further research. The results of antimicrobial studies are summarized in Table 1.

### 2.3. Identification of Antimicrobial Pharmacophore Sites of **1**–**10**

**Figure 1.**
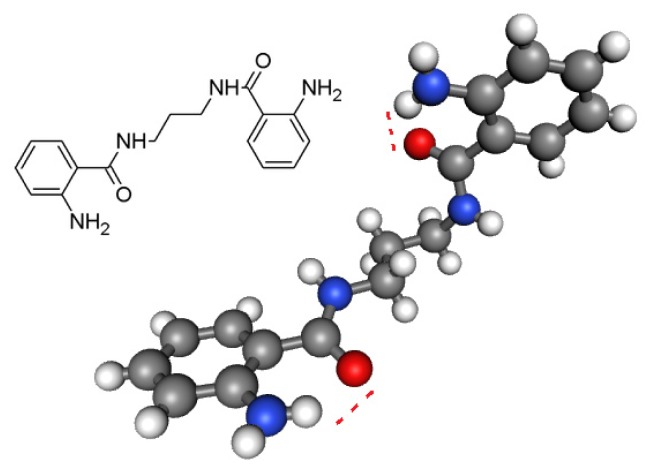
The structural parameters do not indicate a tautomeric equilibrium but a single amino/amido form. The main differences between the two crystalline forms lie in the intramolecular hydrogen of NH_2_ bonding and its relative orientation to oxygen of amide [28]. Attractive intramolecular interactions occur and are responsible for the closure of pharmacophore site (C=O^δ−^–NH_2_^δ+^).

**Figure 2.**
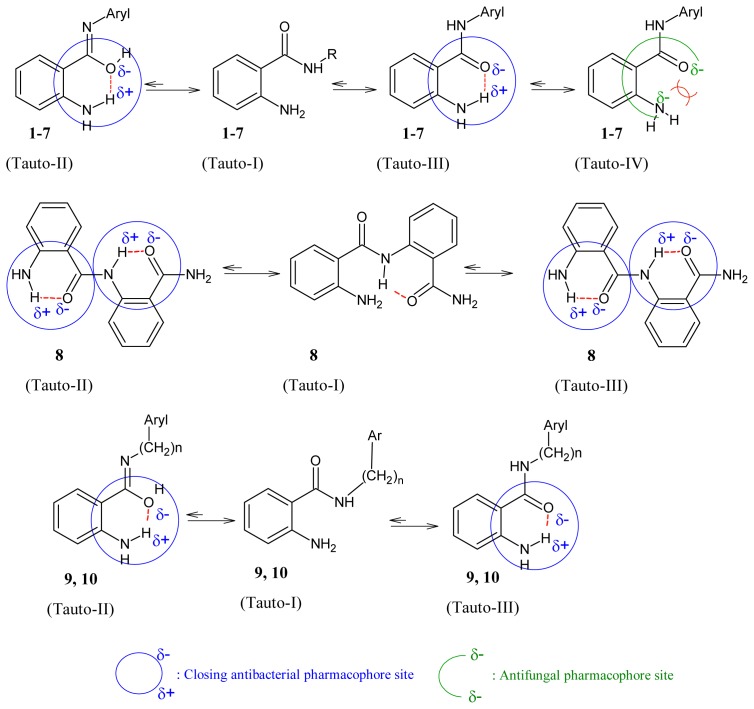
Impact of tautmerism on opening/closing antimicrobial pharmacophore site of **1**–**10**.

It was believed that amide can exist in two major tautomeric forms, keto-amine and hydroxy-enamine, although keto-amine form is predominant in the solid state [28] as shown in Figure 1. We investigated the potential pharmacophores of various bioactive compounds for their antibacterial [29–33], antifungal [34,35] and antiviral activity [36], and verified them further with Petra/Osiris/Molinspiration (POM) analyses. On the basis of our previously published findings in antibacterial, antiviral and antifungal fields, we can safely conclude that our present series, ABD **1**–**10**, also contain a pharmacophore responsible for antifungal and antibacterial activities which is elaborated in Figure 2. It was also hypothesised that the difference in charges between two heteroatoms of the same dipolar pharmacophoric site may facilitate the inhibition of bacteria more than viruses and fungi. For antibacterial activity, the compounds possess (X^δ−^–Y^δ−^) pharmacophore site (II, III) and for antiviral activity the compounds possess (X^δ−^–Y^δ−^) pharmacophore site.

The following crystallographic results (Figure 1) support our hypothesis that 2-aminobenzamide derivatives (**1**–**10**) or any heterocyclic ring present in adjacent position to NH could generate at least four tautomeric forms and apparently two closed pharmacophore sites through the formation of six-membered intramolecular ring assisted by hydrogen bonding, which are responsible for decreasing both antibacterial (C=O^δ−^–^δ+^HN) and antifungal activity (C-OH^δ+^–^δ−^N=C).

## 3. Experimental Section

### 3.1. General

Melting points (M.P.) were measured on a Gallenkamp melting point apparatus in open glass capillaries and are uncorrected. IR spectra were measured as KBr pellets on a Perking Elmer FT 1000 spectrophotometer (Madison, WI, USA). The NMR spectra were recorded on a Varian Mercury Jeol-400 NMR spectrometer (Tokyo, Japan). ^1^H-NMR (400 MHz) and ^13^C-NMR (100 MHz) were run in (DMSO-*d*_6_). Chemical shifts (δ) are referred in ppm and coupling constants *J* are given in Hz. Mass spectra were recorded on a Shimadzu GCMS-QP 1000 EX mass spectrometer (Tokyo, Japan) at 70 eV. Elemental analysis was carried out on an Elementar Vario EL analyzer (Vernon Hills, IL, USA). Sample Availability: Samples of the compounds **1**–**10** are available from the authors.

### 3.2. General Procedure for the Synthesis of Compounds **1**–**10**

#### 3.2.1. Procedure A

To a solution of isatoic anhydride (5–10 mmol, 1.equiv.) in 5–10 mL DMF were added a solution of amine derivatives (5–10 mmol, 1.equiv.) in 5–10 mL DMF and the reaction mixture was refluxed for 6 h. The reaction mixture was monitored by TLC (EtOH:CHCl_3_). After completion of reaction, the reaction mixture was cooled to room temperature. The precipitated solid product formed was then filtered off and recrystallized to afford the respective product.

#### 3.2.2. Procedure B

A mixture of isatoic anhydride (5–10 mmol, 1.equiv.) and amine derivatives (5–10 mmol, 1.equiv.) in the presence of few drops from DMF was exposed to microwave irradiation (140–420 W) for about 4–10 min. The reaction mixture was then cooled to room temperature and ice cooled water (5 mL) was added to the reaction mixture which resulted in the formation of solid precipitates. These precipitates were filtered off and recrystallized to get the pure products.

##### 3.2.2.1. 2-Amino-*N*-(4-fluorophenyl)benzamide (**1**)

Compound **1** was prepared according to method A or method B (10 min,140 W), isolated as brown powder; yield (72**^a^**, 65**^b^** %); mp 122 °C; IR *ν*_max_ (KBr) 3470.3, 3366.19, 3275.55, 1636.53 cm^−1; 1^H-NMR (400 MHz, DMSO-*d*_6_) (ppm): δ 10.04 (1H, br s, NH), 7.71–7.74 (2H, dd), 7.62 (1H, d, *J* = 8.0 Hz), 7.14–7.20 (3H, m), 6.75 (1H, d, *J* = 8.0 Hz), 6.59 (1H, t, *J* = 8.0 Hz), 6.33 (2H, br s, NH_2_);^13^C-NMR: δ 115.3, 115.7, 116.9, 122.9, 129.1, 132.6, 136.1, 168.3; MS *m*/*z* (%): 230 (M**^+^**, 100); Anal. for C_13_H_11_N_2_OF (230.24) calcd; C, 67.82; H, 4.82; N, 12.17. Found: C, 67.81; H, 4.80; N, 12.25.

##### 3.2.2.2. 2-Amino-*N*-(4-chlorophenyl)benzamide (**2**)

Compound **2** was prepared according to method A or method B (5 min, 140 W), isolated as brown powder; yield (80**^a^**, 70**^b^** %); mp 147 °C; IR *ν*_max_ (KBr) 3463, 3364, 3285, 1638 cm^−1; 1^H-NMR (400 MHz, DMSO-*d*_6_) (ppm): δ 10.12 (1H, br s, NH), 7.76 (2H, d, *J* = 8.8 Hz), 7.62 (1H, d, *J* = 7.7 Hz), 7.38 (2H, d, *J* = 8.8 Hz), 7.21 (1H, t, *J* = 7.7 Hz), 6.76 (1H, d, *J* = 7.7 Hz), 6.59 (1H, t, *J* = 7.7 Hz), 6.40 (2H, br s, NH_2_);^13^C-NMR: δ 115.2, 115.4, 116.9, 122.5, 127.5, 128.9, 129.2, 132.8, 138.8, 150.3, 168.4; MS *m*/*z* (%): 246 (M**^+^**, 100) [M^+^] (C_13_H_11_N_2_OCl) (73.85), 216 (6.25), 165 (10.94), 109 (25.00), 74 (43.08), 30 (100); Anal. for C_13_H_11_ClN_2_O (246.69) calcd; C, 63.29; H, 4.49; N, 11.36; Found: C, 63.30; H, 4.51; N, 11.33.

##### 3.2.2.3. 2-[4-(2-Aminobenzamido)phenyl]acetic acid (**3**)

Compound **3** was prepared according to method A or method B (4 min, 280 W) isolated as yellowish green powder; yield (99**^a^**, 60**^b^** %); mp 188.5 °C; IR *ν*_max_ (KBr) 3483, 2600, 1698.08, 1639.97 cm^−1; 1^H-NMR (400 MHz, DMSO-*d*^6^) (ppm): δ 12.38 (1H, br s, OH), 9.98 (1H, br s, NH), 7.62–7.67 (3H, m), 7.18–7.23 (3H, m), 6.76 (1H, d, *J* = 7.7 Hz), 6.59 (1H, t, *J* = 7.7 Hz), 6.36 (2H, br s, NH_2_), 3.54 (2H, s, CH_2_); ^13^C-NMR: δ 40.2, 115.2, 115.8, 116.9, 121.0, 129.2, 129.9, 130.5, 132.6, 138.3, 150.2, 168.3, 173.4; MS *m*/*z* (%): 270 [M^+^] (C_15_H_14_N_2_O_3_) (100), 224 (22.12), 196 (25.74), 150 (56.55), 138 (13.34), 74 (10.12); Anal. for C_15_H_14_N_2_O_3_ (270.28) calcd; C, 66.66; H, 5.22; N, 10.36; Found: 66.67; H, 5.23; N, 10.29.

##### 3.2.2.4. 2-Amino-*N*-(3,4-dimethoxyphenyl)benzamide (**4**)

Compound **4** was prepared according to method A or method B (4 min, 400 W), recrystallized from ethanol and isolated as black powder; yield (65**^a^**, 68**^b^** %); mp 166 °C; IR *ν*_max_ (KBr) 3439, 3332, 3376, 1643 cm^−1; 1^H-NMR (400 MHz, DMSO-*d*_6_) (ppm): δ 9.85 (1H, br s, NH), 7.60 (1H, d, *J* = 8.0 Hz), 7.42 (1H, s), 7.27 (1H, d, *J* = 8.8 Hz), 7.18 (1H, t, *J* = 8.0 Hz), 6.91 (1H, d, *J* = 8.8 Hz), 6.74 (1H, d, *J* = 8.0 Hz), 6.58 (1H, t, *J* = 8.0 Hz), 6.34 (2H, br s, NH_2_), 3.73, 3.74 (3H, s, 2(OCH_3_); ^13^C-NMR: δ 55.9, 56.2, 106.2, 112.3, 113.0, 115.1, 115.9, 116.8, 129.0, 132.4, 133.3, 145.5, 148.9, 150.2, 168.0; MS *m*/*z* (%): 272 [M^+^] (C_15_H_16_N_2_O_3_) (100), 273 [M^+^ + 1] (98.97), 257 (15.62), 241 (12.34), 230 (38.46), 201 (46.92); Anal. for C_15_H_16_N_2_O_3_ (272.30) calcd; C, 66.16; H, 5.92; N, 10.29; Found: C, 66.22; H, 5.88; N, 10.26.

##### 3.2.2.5. 2-Amino-*N*-(4-methoxyphenyl)benzamide (**5**)

Compound **5** was prepared according to method A or method B (4 min, 140 W) and isolated as beige powder; yield (99**^a^**, 84**^b^** %); m.p. 121 °C; IR *ν*_max_ (KBr) 3454, 3366, 3274, 1643 cm^−1; 1^H-NMR (400 MHz, DMSO-*d*_6_) (ppm): δ 7.75 (1H, br s, NH), 7.42–7.44 (3H, m), 7.22 (1H, t, *J* = 7.7 Hz), 6.86–6.88 (2H, m), 6.65–6.69 (2H, m), 5.12 (2H, br s, NH_2_), 3.78 (3H, s, OCH_3_); ^13^C-NMR: δ 55.5, 114.2, 116.3, 116.8, 117.5, 122.7, 127.2, 130.8, 132.6, 148.9, 156.7, 167.6; MS *m*/*z* (%): 242 [M^+^] (C_14_H_14_N_2_O_2_) (80.00), 225 (17.22), 212 (11.34), 194 (100), 77 (37.43), 76 (25.12); Anal. for C_14_H_14_N_2_O_2_ (242.27) calcd; C, 69.41; H, 5.82; N, 11.56; Found: C, 69.43; H, 5.81; N, 11.55.

##### 3.2.2.6. 2-Amino-*N*-(3,4,5-trimethoxyphenyl)benzamide (**6**)

Compound **6** was prepared according to method A and isolated as brown cubes; yield (75**^a^** %); mp 217 °C; IR *ν*_max_ (KBr) 3461, 3345, 3146, 1654 cm^−1; 1^H-NMR (400 MHz, DMSO-*d*_6_) (ppm): δ 9.89 (1H, br s, NH), 7.60 (1H, d, *J* = 7.8 Hz), 7.18–7.22 (3H, m), 6.75 (1H, d, *J* = 7.8 Hz), 6.59 (1H, t, *J* = 7.8 Hz), 6.35 (2H, br s, NH_2_), 3.76 (6H, s, 2(OCH_3_*)), 3.64 (3H, s, OCH_3_); ^13^C-NMR: δ 56.2, 60.6, 98.6, 115.1, 115.7, 116.9, 129.0, 132.6, 134.0, 135.9, 150.2, 153.0, 168.2; MS *m*/*z* (%): 302 [M^+^] (C_16_H_18_N_2_O_4_) Anal. for C_16_H_18_N_2_O_4_ (302.33) calcd; C, 63.56; H, 6.00; N, 9.27; Found: C, 63.59; H, 6.03; N, 9.29.

##### 3.2.2.7. 2-Amino-*N*-(p-tolyl)benzamide (**7**)

Compound **7** was prepared according to method A, recrystallized from benzene and isolated as beige powder; yield (97**^a^** %); mp 149 °C; IR *ν*_max_ (KBr) 3464.73, 3361.59, 3273.20, 1636.80 cm^−1; 1^H-NMR (400 MHz, DMSO-*d*_6_) (ppm): δ 7.76 (1H, br s, NH), 7.41–7.45 (3H, m), 7.23 (1H, t, *J* = 7.7 Hz), 7.15 (2H, d, *J* = 8.0 Hz), 6.68–6.70 (2H, m), 5.43 (2H, br s, NH_2_), 2.33 (3H, s, CH_3_); ^13^C-NMR: δ 21.0, 116.4, 116.8, 117.5, 120.8, 127.3, 129.6, 132.7, 134.2, 135.3, 148.9, 167.6; MS *m*/*z* (%): 226 [M^+^] (C_14_H_14_N_2_O) (31.25), 209 (92.31), 191 (15.62), 133 (29.69), 118 (100), 105 (31.25); Anal. for C_14_H_14_N_2_O (226.27) calcd; C, 74.31; H, 6.24; N, 12.38; Found: 74.29; H, 6.25; N, 12.37.

##### 3.2.2.8. 2-Amino-*N*-(2-carbamoylphenyl)benzamide (**8**)

Compound **8** was prepared according to method A or method B (6 min, 280 W) and isolated as beige powder; yield (60**^a^**, 64**^b^** %); m.p. 110 °C; IR *ν*_max_ (KBr) 3411, 3325, 3187, 1660, 1618 cm^−1; 1^H-NMR (400 MHz, DMSO-*d*_6_) (ppm): 1.73 (1H, br s, NH), 7.91 (1H, d, *J* = 7.5 Hz, H-2′), 7.73 (1H, t, *J* = 7.5 Hz, H-4′), 7.52 (1H, d, *J* = 7.5 Hz, H-5′), 7.24 (1H, t, *J* = 7.5 Hz, H-3′), 7.10–7.16 (2H, m, H-4, 6), 6.67 (1H, d, *J* = 7.7 Hz, H-3), 7.59, 7.05 (each 2H, s, 2(NH_2_)), 6.47 (1H, t, *J* = 7.7 Hz, H-5); ^13^C-NMR: δ 110.79, 114.22, 114.91, 115.88, 116.94, 124.05, 129.29, 129.48, 132.43, 137.47, 141.93, 147.65, 160.43, 171.85; MS *m*/*z* (%): 255.27 [M^+^] (C_14_H_13_N_3_O_2_]; Anal. for C_14_H_13_N_3_O_2_ (255.27) calcd; C, 65.87; H, 5.13; N, 16.46; Found: C, 65.90; H, 5.11; N, 16.45.

##### 3.2.2.9. 2-Amino-*N*-(4-chlorobenzyl)benzamide (**9**)

Compound **9** was prepared according to method A or method B (10 min, 280 W), recrystallized from methanol and isolated as white powder; yield (95**^a^**, 62**^b^** %); mp 137 °C; IR *ν*_max_ (KBr) 3472, 3358, 3308, 1638 cm^−1; 1^H-NMR (400 MHz, DMSO-*d*_6_) (ppm): δ 7.55 (1H, d, *J* = 8.0 Hz), 7.38 (2H, d, *J* = 8.0 Hz), 7.33 (2H, d, *J* = 8.0 Hz), 7.14 (1H, t, *J* = 8.0 Hz), 6.70 (1H, d, *J* = 8.0 Hz), 6.52 (1H, t, *J* = 8.0 Hz), 6.45 (2H, br s, NH_2_), 4.40 (2H, s, CH_2_); ^13^C-NMR: δ 40.2, 114.7, 115.1, 116.9, 128.6, 128.7, 129.5, 131.7, 132.4, 139.5, 150.3, 169.4; MS *m*/*z* (%): 260 [M^+^] (C_14_H_13_N_2_OCl) (86.22), 261 [M^+^ + 1] (100), 229 (21.33), 225 (32.40), 214 (28.86), 192 (65.90); Anal. for C_14_H_13_ClN_2_O (260.72) calcd; C, 64.49; H, 5.03; N, 10.74; Found: C, 64.53; H, 5.08; N, 10.68.

##### 3.2.2.10. 2-Amino-*N*-(3,4-dimethoxyphenethyl)benzamide (**10**)

Compound **10** was prepared according to method A and isolated as beige powder; yield (100**^a^** %); mp 105 °C; IR *ν*_max_ (KBr) 3326, 3415, 1637 cm^−1; 1^H-NMR (400 MHz, DMSO-*d*_6_) (ppm): δ 8.26 (1H, br s, NH), 7.44 (1H, d, 7.5 Hz), 7.12 (1H, t, *J* = 7.5 Hz), 6.82–6.87 (2H, m), 6.73–6.76 (1H, dd, *J* = 7.5 Hz), 6.69 (1H, d, *J* = 8.0 Hz), 6.50 (1H, t, *J* = 7.5 Hz), 6.40 (2H, br s, NH_2_), 3.72–3.71 (each 3H, s, 2(OCH_3_)), 3.41 (2H, t, *J* = 6.9 Hz, CH_2_), 2.76 (2H, t, *J* = 7.3 Hz,CH_2_); ^13^C- NMR: δ 39.8, 40.4, 55.8, 56.0, 112.3, 113.0, 115.0, 115.5, 116.8, 121.0, 28.5, 132.1, 132.6, 147.7, 149.1, 150.1, 169.3; MS *m*/*z* (%): 300 [M^+^] (C_17_H_20_N_2_O_3_) 301 [M^+^ + 1] (100), 302 [M^+^ + 2] (21.33), 287 (4.12), 184 (10.62), 179 (12.10); Anal. for C_17_H_20_N_2_O_3_ (300.35) calcd; C, 67.98; H, 6.71; N, 9.33; Found: C, 68.00; H, 6.72; N, 9.38.

## 4. Antifungal Activity

Tested samples were screened separately *in vitro* for their antifungal activity various fungi viz. *Aspergillus fumigatus* (RCMB 002003), *Saccharomyces cerevislae* (RCMB 006002), and *Candida albicans* (RCMB 005002) . The culture of fungi was purified by a single spore isolation technique. The antifungal activity was by an agar well diffusion method by the following procedure.

Sabourad dextrose agar plates: A homogeneous mixture of glucose-peptone-agar (40:10:15) was sterilized by autoclaving at 121 °C for 20 min. The sterilized solution (25 mL) was poured into each sterilized petri dish in laminar flow and left for 20 min to form the solidified sabourad dextrose agar plate. These plates were inverted and kept at 30 °C in incubator to remove the moisture and to check for the contamination.

Fungal strain was grown in 5 mL sabourad dextrose broth (glucose: peptone; 40:10) for 3–4 days to achieve 105 CFU/mL cells. The fungal culture (0.1 mL) was spread out uniformly on the sabourad dextrose agar plates by sterilized triangular folded glass rod. Plates were left for 5–10 min so that culture is properly adsorbed on the surface of sabourad dextrose agar plates. Now, small wells of size 4 mm × 2 mm were cut into the plates with the help of a well cutter, and the bottom of the wells were sealed with 0.8% soft agar to prevent the flow of test sample at the bottom of the well. One hundred μL of the tested samples (10 mg/mL) were loaded into the wells of the plates. All compounds were prepared in dimethyl sulfoxide, DMSO was loaded as control. The plates were kept for incubation at 30 °C for 3–4 days and then the plates were examined for the formation of a zone of inhibition. Each inhibition zone was measured three times by caliper to get an average value. The test was performed three times for each fungus. Clotrimazole was used as antifungal standard drug.

## 5. Antibacterial Activity

Antibacterial activities were investigated using an agar well diffusion method. The activity of tested samples was studied against the *Staphylococcus aureus* (RCMB 000106) and *Bacillis subtilis* (RCMB 000107), as Gram positive bacteria and *Pseudomonas aeruginosa* (RCMB 000102) and *Escherichia coli* (RCMB 000103), as Gram negative bacteria. The solution of 5 mg/mL of each compound in DMSO was prepared for testing against bacteria. Centrifuged pellets of bacteria from 24 h old culture containing approximately 104–106 CFU (colony forming unit) per ml were spread on the surface of nutrient agar (typetone 1%, yeast extract 0.5%, NaCl 0.5%, agar 1000 mL of distilled water, PH 7.0) which was autoclaved under 121 °C for at least 20 min. Wells were created in medium with the help of sterile metallic bores and then cooled down to 45 °C. The activity was determined by measuring the diameter of the inhibition zone (in mm). One hundred μL of the tested samples (10 mg/mL) were loaded into the wells of the plates. All compounds was prepared in DMSO, DMSO was loaded as control. The plates were kept for incubation at 37 °C for 24 h and then the plates were examined for the formation of a zone of inhibition. Each inhibition zone was measured three times by caliper to get an average value. The test was performed three times for each bacterium. Streptomycin was used as antibacterial standard drug.

## 6. Conclusions

In conclusion, we have successfully prepared 2-Aminobenzamide derivatives **1**–**10** starting from isatoic anhydride ISA and reacting it with appropriate *N*-nucleophile. A simple, mild, time efficient, high yielding and environmentally friendly microwave irradiation procedure has been introduced in addition to a classical method for the synthesis of benzamide derivatives. Furthermore, the antimicrobial potential of the selected synthesized compounds was also evaluated. Compound **5** showed excellent antimicrobial potential against all tested bacterial and fungal strains. However, it was found more potent than the standard drug against *Aspergillus fumigatus* species. All other compounds exhibited moderate to good activity against one or more tested strains of bacteria and fungi.

## Figures and Tables

**Table 1.** Antimicrobial activity (inhibition zones in mm) of some of compounds **1**–**10** [e].

Compound	Bacteria				Fungi		
	
	(A) [a]	(B) [a]	(C) [b]	(D) [b]	(E)	(F)	(G)
**1**	9.9	9.7	9.1	9.4	11.8	11.2	11.9
**2**	7.2	7.8	7.4	6.9	9.4	8.2	8.4
**3**	11.9	11.1	10.4	9.5	11.8	11.5	11.2
**5**	17.9	19.2	10.9	12.4	19.7	20.1	15.8
**7**	9.1	8.8	7.9	7.6	12.2	9.4	7.2
**CLOZO** [c]	-	-	-	-	18.3	23.1	26.1
**STREPT** [d]	25.1	30.1	25.6	24.3	-	-	-

(A): *Staphylococcus aureus* (RCMB 000106); (B): *Bacillis subtilis* (RCMB 000107); (C): *Pseudomonas aeruginosa* (RCMB 000102); (D): *Escherichia coli* (RCMB 000103); (E): *Aspergillus fumigatus* (RCMB 002003); (F): *Saccharomyces cerevislae* (RCMB 006002); (G): *Candida albicans* (RCMB 005002);

[a] Antibacterial Gram-positive;

[b] Antibacterial Gram-negative;

[c] CLOZO: Clotrimazole (St. 25 μL/mL);

[d] STREPT; Streptomycin (St. 25 μL/mL);

[e] Mean zone of inhibition in mm ± standard deviation beyond well diameter (6 mm) produced on a range of environmental and clinically pathogenic microorganism using (5 mg/mL) concentration of tested samples.
